# Maternal and fetal ultrasonography, vulvar temperature and vaginal mucous impedance for the prediction of parturition in Saanen does

**DOI:** 10.1590/1984-3143-AR2023-0006

**Published:** 2023-04-21

**Authors:** Priscila Del’Aguila-Silva, Fabiana Cirino dos Santos, Luiz Paulo Nogueira Aires, Ricardo Andres Ramirez Uscategui, Lizandra Amoroso, Wilter Ricardo Russiano Vicente, Marcus Antônio Rossi Feliciano

**Affiliations:** 1 Departamento de Clínica e Cirurgia Veterinária, Faculdade de Ciências Agrárias e Veterinárias, Universidade Estadual Paulista “Júlio de Mesquita Filho”, Jaboticabal, SP, Brasil; 2 Departamento de Morfologia e Fisiologia Animal, Faculdade de Ciências Agrárias e Veterinárias, Universidade Estadual Paulista “Júlio de Mesquita Filho”, Jaboticabal, SP, Brasil; 3 Grupo de investigación INCA-CES, Facultad de Medicina Veterinaria y Zootecnia, Universidad CES, Medellín, Colombia; 4 Departamento de Medicina Veterinária, Faculdade de Zootecnia e Engenharia de Alimentos, Universidade de São Paulo, Pirassununga, SP, Brasil

**Keywords:** echobiometric, caprine, pregnancy

## Abstract

The aim of this study was to evaluate and correlate modifications of vaginal mucous impedance, vulvar temperature and ultrasonographic measurements (echobiometric parameters) to parturition in pregnant Saanen does. 30 does were selected for the study and submitted to an estrus synchronization protocol and natural mating. The females were evaluated daily from Day 143 of pregnancy to parturition. For the sonographic evaluations, the following structures were measured: biparietal diameter, thoracic diameter, abdominal diameter, ocular orbit, kidney length, kidney height, cardiac area, placentome length, cervical measurement and fetal heart rate; by means of two different approaches: transrectal and transabdominal, using a 7.5 MHz linear transducer. The vaginal mucous impedance was assessed using an electric estrous detector and vulvar temperature was measured using a non-contact infrared thermometer. Statistical analysis was performed using the R-project software and the significance level was set at 5% for all tests. 25 Saanen does became pregnant, resulting in 80.33% pregnancy rate. Fetal heart rate was negatively correlated to the hours to parturition (p<0,001; r-Pearson= -0,451), as well as vaginal temperature (p= 0,001; r-Pearson= -0,275), while cervical thickness was positively correlated to hours to parturition (p<0,001; r-Pearson= 0,490). The echobiometric parameters (biparietal diameter, thoracic diameter, abdominal diameter, ocular orbit, kidney length and height, cardiac area, placentome length), as well as vaginal mucous impedance did not vary throughout the timepoints of evaluation and did not correlate to the moment of parturition. It was concluded that the parameters of fetal heartbeat, vaginal temperature and cervical effacement in the last week of pregnancy provide valuable information regarding the proximity of parturition.

## Introduction

Ultrasonographic gestational diagnosis and monitoring of pregnancy is essential for characterization of embryonic and fetal development, as well as for the identification of any problems related to pregnancy, estimation of gestational age, in addition to fetal counting and sexing in goat herds. Accurate information regarding pregnancy stage is also useful for monitor females close to parturition, contributing to the verification of fetal viability and decision-making in cases of dystocia (Doize et al., 1997; González De Bulnes et al., 1998).

Parturition is the process of delivery of the fully-grown fetus and it is a crucial step in small ruminant production, as the prevalence of dystocia in these species is relatively high and the global neonatal mortality rate varies from 10 to 30% (Dwyer, 2008). Modifications that occur directly in the cervix are important in controlling the parturition process, there is a cervical preparation and the tissue becomes distensible and soft for the passage of the conceptus (Mesiano et al., 2014). Another sign that may indicate proximity to the parturition process is the decrease in body temperature (Burfeind et al., 2011).

The visualization of the intestines and the identification of peristalsis by ultrasound determine the end of fetal organogenesis in small animals, indirectly indicating that the fetus is at term (Gil et al., 2015), as well as acceleration and deceleration occur in canine fetuses and predicted the optimal time of parturition (Gil et al., 2014). According to Giannico et al. (2015), changes in umbilical blood flow, detected with pulsed wave Doppler imaging, may be predictive of the time of parturition as well as potential fetal distress in pregnant bitches.

In small ruminants, increases in plasma concentrations of cortisol induces the biosynthesis of estrogen relative to progesterone and this change in E:P ratio increases myometrial activity and culminates in labor and parturition (Wood, 1999). In ewes, some studies demonstrate the correlation between the electric resistance of the vaginal mucosa to serum concentration of estradiol and progesterone during the estrous cycle (Bartlewski et al., 1999; Theodosiadou and Tsiligianni, 2015). Considering that it is possible to relate the concentration of these hormones to estrus detection, measuring the electric resistance of the vaginal mucosa could be useful in detecting the proximity of parturition.

Understanding the mechanisms that regulate parturition and the acknowledgment of its proximity can aid the implementation of interventional procedures to avoid or reduce perinatal morbidity and mortality. To the present date, no studies were found in literature which have tried to correlate vaginal mucous impedance of does to the moment of parturition, as well as certain echobiometric parameters and temperature variations in the peripartum period. The aim of this study was to evaluate and compare modifications in vaginal mucous impedance, vulvar temperature and echobiometric measurements to the moment of parturition in the caprine species.

## Methods

### Animals

This research was approved by the Ethics Committee on Animal Use of Universidade Estadual Paulista (protocol nº 010229/17), located in Jaboticabal, Sao Paulo, Brazil (latitude: 21◦15’18”S, longitude 48◦19’19”W). A total of 30 (thirty) healthy adult (2 - 5 years of age) pluriparous Saanen does, weighing 55 ± 10 kg were included in this study. Each patient was considered healthy based on physical and obstetric examination and none presented history of reproductive diseases.

The diet of the subjects was consisted of corn silage (70% inclusion) offered *ad libitium* adjusted to maintain from 15 to 20% leftovers of the offered amount and concentrate based on corn bran, soy bran, ground soy grain and wheat bran (30% inclusion). Food was offered twice daily (at 8 a.m. and 4 p.m.) and commercial mineral salt (Caprinofós®, DSM Nutritional Products, Campinas, SP, Brazil) was offered separately (30 g/animal/day).

### Estrous synchronization and mating

After selection of the subjects, each female was subjected to a short estrus synchronization protocol at different time points. This protocol was consisted of placement of an intravaginal device containing 0.33 g of progesterone (Eazi breed CIDR®, Pfizer, New Zealand) on Day 0 (random day of the cycle), followed by intramuscular administration of 37.5 µg of cloprostenol (Sincrocio®, Ourofino, Cravinhos, SP, Brazil) and 300 UI of equine chorionic gonadotropin (Novormon® Coopers MSD, Brazil) on Day 5 and removal of the implant device on Day 6. On Day 7, the females were allocated with a buck for 24 hours to ensure the exact day of conception.

### Ultrasound evaluation

For sonographic evaluation, the patients were kept in a quadrupedal position and the examinations were performed with a MyLab-30-VET equipment (Esaote, Genova, Italy). Evaluations were performed in two different approaches. For the transrectal approach, a specific 7.5 MHz linear transrectal transducer. Feces were removed from the rectum prior to the evaluation and an acoustic coupling ultrasound gel was used. For the transabdominal approach, a linear 7.5 MHz linear transducer was used. Abdominal and inguinal hair was clipped prior to the sonographic assessment and an acoustic coupling ultrasound gel was used for optimal image acquisition.

On Day 21 after mating, ultrasound evaluation was performed on the patients for pregnancy diagnosis using the transrectal approach due to the small size of the uterus and its location in the pelvic floor in this period. Criteria for pregnancy diagnosis at this stage were the visualization of anechoic areas on the uterine lumen (embryonic vesicle), embryo, or placentomes (Karadaev et al., 2018). The urinary bladder was used as an anatomical landmark for the identification and evaluation of the reproductive tract.

After the confirmation of pregnancy, daily sonographic evaluations were performed from Day 143 of pregnancy until parturition. Fetal echobiometry was obtained by the transabdominal ultrasonographic approach and, specifically for the evaluation of the cervix, the examination was performed using the transrectal approach.

For the echobiometric parameters, the following structures were measured (in mm, with the exception of the cardiac area, which was obtained in cm^2^): biparietal diameter (BPD), thoracic diameter (TC), abdominal diameter (AD), ocular orbit (OO), kidney length (KL), kidney height (KH) (in longitudinal view), cardiac area (CA) (in transverse view), placentome length (PL), cervical measurement (CM) and fetal heart rate (FHR), this last parameter evaluated using Pulsed-wave Doppler.

In all evaluations, only one conceptus was assessed, regardless of singleton or multiple pregnancy. However, the number of conceptuses was always recorded, as with the progression of the pregnancy, it was not possible to evaluate multiple conceptuses simultaneously and it was not possible to assure that one of the conceptuses was not evaluated more than once.

### Vaginal mucous impedance and vulvar temperature

Vaginal mucosa impedance was assessed using an electric estrous detector (Draminsk® S.A. Olsztyn, Poland). The probe was inserted in the vagina of each doe until end of the shaft met the cervix. The probe was then swirled for better contact of the electrodes at the end of the shaft for adequate reading. Three consecutive readings were obtained before the probe was removed from the vagina.

After impedance readings, vulvar temperature was measured in triplicate using a non-contact infrared thermometer (Incoterm®, Avita/Wujiang Co., Ltd - Wu Jiang/China) aimed 4 - 6cm away from the vulva after careful retraction of the labia.

### Parturition and neonatal evaluation

Considering the data regarding the date of mating and gestational age, in the last week of pregnancy, the patients were evaluated daily for monitoring of the peripartum period.

After birth, the neonates were evaluated regarding neonatal viability with the aid of the Apgar score described by Vannucchi et al. (2012), immediately after birth, 15 minutes and 60 minutes after birth. A score from 0 to 2 was assigned for each of the following parameters: heart rate and respiratory rate evaluated during one minute, mucosal color and muscle tonus tone through inspection and reflex irritability to pinching in the interdigital space. Additionally, the body temperature and weight of all animals were measured. In addition, the animals born were monitored for their body development through weighing every 15 days until 120 days of age.

### Statistical analysis

Statistical analysis was performed using the R-project software (R® foundation for statistical computing, Austria). Initially, the distribution of variables was tested (Shapiro test). Subsequently, the real or transformed measurements were correlated with the hours to parturition (retrospectively) by the Spearman test. When significant, the adjustments of variables (separately and in groups) were then tested as predictors of hours before parturition (HBP) as responses using regression models (linear, quadratic and cubic). For these same variables that were significantly correlated with HBP, ROC (receiver-operating characteristic) curves were analyzed using the 24 hours before parturition as a reference for calculating the cut-off point, sensitivity and specificity to predict parturition. The significance level was set at 5% for all tests and the results presented as the mean ± SD (standard deviation).

## Results

Of the 30 does selected for the study, 25 became pregnant after the synchronization protocol and allocation with a buck, resulting in 80.33% pregnancy rate. Of all pregnancies in this study, 5 were singletons (20%), 15 were twin (60%), 4 were triplet (16%) and 1 was quintuplet (4%), totaling 52 goatlings. Of all goatlings, 48 (92%) were considered healthy based on the Apgar score evaluation (Vannucchi et al., 2012). One of the twin pregnancies resulted in two stillborn goatlings due to maternal dystocia. One of the goatlings from the quintuplet pregnancy presented poor Apgar score during the first hours of evaluation and did not survive after 24 hours of birth.

**Figure 1:**
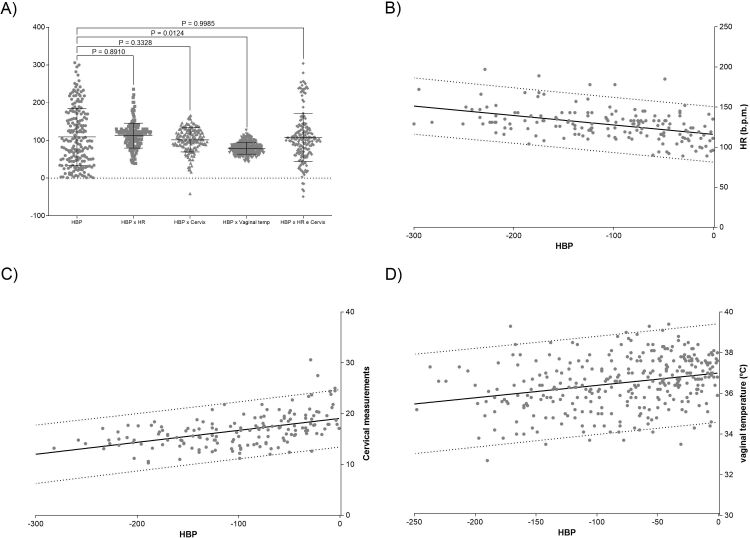
Graphical representation of the trajectory of the parameters evaluated in the hours before parturition (HBP) in Saanen goats evaluated by B-mode ultrasonography. The solid lines represent the regression model adapted for each parameter. Dotted lines indicate the prediction range at 95% confidence. A) Comparison between the parameters. B) Heart rate (HR). C) Cervical measurements. D) Vaginal temperature.

**Figure 2:**
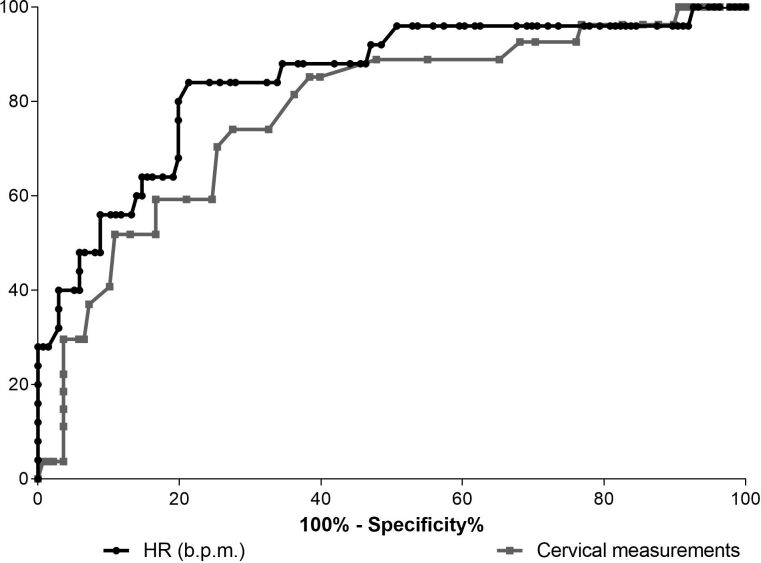
Graphical representation of ROC curves for sensitivity and specificity of fetal heart rate (HR) and opening of the cervix in the hours before parturition.

The modifications of the variables evaluated during the last week of pregnancy are summarized in Figure 1. Fetal heart rate was negatively correlated to hours before parturition (p<0,001; r-Pearson= -0,451), as well as vaginal temperature (p= 0,001; r-Pearson= -0,275). Conversely, cervical thickness was positively correlated (p<0,001; r-Pearson= 0,490) to hours before parturition. Predictive values of these parameters are described in Table 1 and Figure 2.

**Table 1:** Predictive values of fetal heart rate and cervical measurements 24 hours prior to parturition.

**Variables**	**P-Value**	**Cut-off Value**	**Sensitivity**	**Specificity**	**AUC**	**Predictive Equation**	**R^2^**
FHR	<0,001	<122 bpm	70,40%	74.60%	77.90%	HBP = - 123 + 1.82 x FHR	20%
CM	<0,001	>17.3 mm	84,00%	72.80%	84.50%	HBP = 271 - 10.2 x CM	24%

FHR: fetal heart rate; CM: cervical measurement; HBP: hours before parturition; AUC: area under the curve; R^2^: determination coefficient. bpm: beats per minute; mm: millimeters.

**Figure 3:**
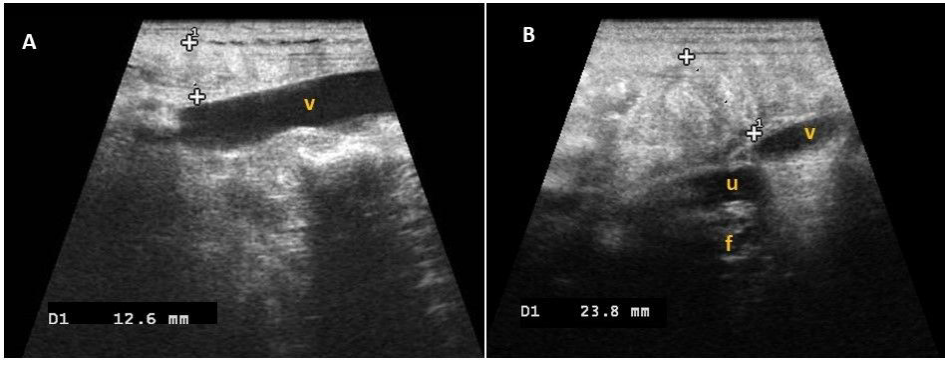
B-mode ultrasound images of the cervix of pregnant goats evaluated transrectally in the peripartum period. A: Cervical measurement (between cursors) on day 145 of pregnancy with visualization of the urinary vesicle (v). B: Cervical measurement (between cursors) on day 150 of gestation with visualization of the urinary vesicle (v), uterus (u) and fetal head (f).

Additionally, qualitative sonographic assessment of the cervix demonstrated a decrease of the classical wrinkling pattern and, when very close to parturition, it was possible to identify the presence of mucus/fluid in the cervical lumen (Figure 3).

Conversely, echobiometric parameters of BPD, TD, AD, OO, PL, KL, KH and CA, as well as vaginal mucous impedance did not vary throughout the different time points of evaluation and did not correlate to hours to parturition.

## Discussion

The evaluations proposed were easily performed, feasible and presented no deleterious effects to the females or the fetuses, considering the only cases of stillborn goatlings and neonatal death did not have any relation to the procedures performed in this study, but rather maternal dystocia and respiratory distress probably due to poor pulmonary maturation. The results of this study provide relevant and unprecedented information regarding the modification of the variables studies close to parturition in goats. The cut-off values determined for fetal heart rate and cervical measurements can provide reliable information regarding the proximity of the parturition moment, aiding the obstetric management of these patients, especially in cases where complications might occur and interventional procedures would be required.

The fetal heart rate cutoff value established in our study is useful for monitoring both the proximity of delivery within 24 hours and fetal viability with adequate accuracy. The decrease in FHR observed in this study can be explained by the ontogenic increase in arterial blood pressure and decrease of heart rate as reflex, as reported by Unno et al. (1999) in sheep, who obtained a variation from 178 ± 2 beats/min on day 120 to 143 ± 2 beats/min on day 140 during the final gestational period, which can be considered an indication of fetal maturity and proximity to parturition. Unlike what was previously reported in dogs (Gil et al., 2014), in our study it was not possible to observe moments of fetal cardiac acceleration and deceleration in the peripartum period, probably due to the fact that our frequency monitoring was carried out in a reduced period, about 1 to 2 min of evaluation.

The onset of parturition is accompanied by a gradual softening followed by a very rapid cervical dilation during active labor and several endocrine factors regulate the interactions of cervical ultrastructural elements, mediating the extensively dynamic remodeling processes in this structure. The sonographic measurement of cervical effacement in our study allowed monitoring its relaxation and opening when close to parturition, providing a useful parameter to predict the moment of parturition. In addition, ultrasound monitoring of the cervix during pregnancy has already demonstrated that abnormal malleability can lead to excessive shortening or deformation of the canal, causing early disruption of the cervical barrier and consequently premature birth (Peralta et al., 2015). Therefore, the detection of this abnormal malleability of the cervix through ultrasound may be a parameter of preterm birth in women and animals.

In pregnant dairy cows, vaginal mucous impedance decreases from 150 Ohm on day 27 to 120 Ohm on day 10 before parturition and it was maintained until delivery (Schindler et al., 1990). Therefore, similar to the present study, no bioelectric resistance changes of the vaginal mucous were observed on the week prior to parturition. This could me due to the fact that the evaluations were initiated after triggering period of the change in steroidogenesis, considering that this modification is triggered by the increase in fetal cortisol secretion, which starts 20 - 25 days prior to delivery (Bartlewski et al., 1999).

The decrease in body temperature with the proximity of parturition observed in our study is also present in cows (Burfeind et al., 2011). Previous studies demonstrated that these changes in body temperature are associated with the modifications of plasma progesterone concentration during pregnancy, which seems to have a thermogenic role and during the prepartum period, where a decrease in its concentration can be evidenced, body temperature also decreases (Schindler et al., 1990; Suthar et al., 2012). Therefore, jointly with other signs, the decrease in body and vulvar temperatures can be considered indicative of the proximity to parturition.

Althought many maternal and fetal echobiometric measurements taken throughout pregnancy are considered excellent gestational age indicators in Saanen goats (Del'Aguila-Silva et al., 2021) the lack of variation in these measurements in our study was expected, considering that fetal growth was completed in the period in which evaluations were performed, not being possible to correlate these with the moment of parturition. However, some cut-off values that indicate parturition within 24 hours were considered adequate and are indicated for use in the field routine.

## Conclusion

Monitoring fetal heart rate, vaginal temperature and cervical effacement during the last week of pregnancy provides adequate information regarding the proximity of parturition in the caprine species. Conversely, echobiometric measurements and vaginal mucous impedance are not reliable indicators of parturition.
